# Type II PI4-kinases control Weibel-Palade body biogenesis and von Willebrand factor structure in human endothelial cells

**DOI:** 10.1242/jcs.187864

**Published:** 2016-05-15

**Authors:** Mafalda Lopes da Silva, Marie N. O'Connor, Janos Kriston-Vizi, Ian J. White, Raya Al-Shawi, J. Paul Simons, Julia Mössinger, Volker Haucke, Daniel F. Cutler

**Affiliations:** 1 Endothelial Cell Biology Laboratory, University College London, London WC1E 6BT, UK; 2 Bioinformatics Image Core, University College London, London WC1E 6BT, UK; 3 Electron Microscopy Core, MRC Laboratory of Molecular Cell Biology, University College London, London WC1E 6BT, UK; 4 Royal Free Centre for Biomedical Science, andWolfson Drug Discovery Unit, Centre for Amyloidosis and Acute Phase Proteins, Division of Medicine, University College London, London NW3 2PF, UK; 5 Leibniz Institut für Molekulare Pharmakologie (FMP), Molecular Physiology and Cell Biology, Robert-Roessle-Str. 10, 13125 Berlin; 6 Fachbereich Biologie, Chemie, Pharmazie, Freie Universität Berlin, Königin-Luise-Str. 2+4, 14195 Berlin, Germany

**Keywords:** Phosphatidylinositol 4-kinase alpha, Phosphatidylinositol 4-kinase beta, Weibel-Palade body, Von Willebrand factor

## Abstract

Weibel-Palade bodies (WPBs) are endothelial storage organelles that mediate the release of molecules involved in thrombosis, inflammation and angiogenesis, including the pro-thrombotic glycoprotein von Willebrand factor (VWF). Although many protein components required for WPB formation and function have been identified, the role of lipids is almost unknown. We examined two key phosphatidylinositol kinases that control phosphatidylinositol 4-phosphate levels at the trans-Golgi network, the site of WPB biogenesis. RNA interference of the type II phosphatidylinositol 4-kinases PI4KIIα and PI4KIIβ in primary human endothelial cells leads to formation of an increased proportion of short WPB with perturbed packing of VWF, as exemplified by increased exposure of antibody-binding sites. When stimulated with histamine, these cells release normal levels of VWF yet, under flow, form very few platelet-catching VWF strings. In PI4KIIα-deficient mice, immuno-microscopy revealed that VWF packaging is also perturbed and these mice exhibit increased blood loss after tail cut compared to controls. This is the first demonstration that lipid kinases can control the biosynthesis of VWF and the formation of WPBs that are capable of full haemostatic function.

## Abstract (precis)

**Summary:** Phosphatidylinositol 4 kinases regulate the formation and function of an endothelial secretory organelle that is crucial for haemostasis in mammals.

## INTRODUCTION

Endothelial cells line the inner layer of all blood vessels and play a crucial part in regulating vessel formation, function and structure. They are adaptable cells that can quickly respond to stimuli in the blood or tissue to control vessel tone, haemostasis and immune responses. Endothelial cells are characterised by the presence of large rod-shaped secretory organelles called Weibel-Palade bodies (WPBs), which store a variety of factors essential to their function that can be quickly released into the vessel lumen in response to stimuli. The primary cargo of WPBs is von Willebrand factor (VWF), a highly multimerised glycoprotein. Exocytosis of WPB cargo results in unfurling of VWF tubules into long strings, exposing platelet-binding sites and serving to tether platelets to sites of vascular injury, a fundamental step in primary haemostasis. In von Willebrand disease, mutations in VWF can perturb formation of WPBs and affect the release of VWF and the string formation, leading to excessive bleeding (Michaux et al., 2003; Sadler, 1998; Valentijn and Eikenboom, 2013), thus correct biogenesis of WPBs and storage of VWF is crucial for normal haemostasis.

A key phase in establishing a fully functional WPB is its initial formation at the trans-Golgi network (TGN), where the lumenal milieu supports the commencement of VWF multimerisation, pro-protein cleavage and tubulation within the TGN lumen (Sadler, 2009). These processes are essential to the recruitment of other WPB cargo – such as P-selectin, which binds to correctly folded VWF (Lui-Roberts et al., 2005), and the cytoplasmic machinery, such as the clathrin adaptor protein 1 (AP-1) and clathrin itself, which are required for early WPB formation (Lui-Roberts et al., 2005). Less well-understood proteins such as aftiphilin and γ-synergin are also involved, and affect the secretory pathway taken by WPBs (Lui-Roberts et al., 2008). Other proteins essential to later stages of the formation, maturation, transport and exocytosis of WPBs are still being identified (Nightingale and Cutler, 2013), and a highly complex process involving multiple protein complexes at different stages of the life-cycle of this organelle is now being revealed.

In contrast to this rapidly expanding set of protein machinery, the contribution of lipids to WPB formation and function is poorly understood. Phosphoinositides (PIs) are minor components of eukaryotic cell membranes – yet essential to the regulation of diverse cellular processes, including signal transduction, vesicle trafficking, cytoskeletal organisation and platelet function (Liu and Bankaitis, 2010; Min and Abrams, 2013). Through their restricted membrane distribution PIs can act as the lipid determinants of membrane identity, supporting localised recruitment of proteins through coincidence detection (Behnia and Munro, 2005; Carlton and Cullen, 2005). At the TGN, PI 4-phosphate (PI4P) is the predominant PI (Balla and Balla, 2006) and has been shown, together with the ADP-ribosylation factor Arf-1, to recruit AP-1, thereby facilitating formation of clathrin-coated vesicles and exocytic carriers (D'Angelo et al., 2007; Demmel et al., 2008; Duncan and Payne, 2003; Hanada et al., 2007; Levine and Munro, 2002; Mills et al., 2003; Wang et al., 2007, 2003). Generation of PI4P at the TGN in mammals is regulated primarily by the type II PI4-kinases PI4KIIα and PI4KIIβ (Balla and Balla, 2006). Studies in various mammalian cell lines have shown a role for either PI4KIIα (Wang et al., 2003) or PI4KIIβ (Craige et al., 2008; Wieffer et al., 2013) in mediating AP-1 recruitment to the TGN. The single PI4KII enzyme in *Drosophila* has been shown to be required for trafficking of secretory granule proteins (Burgess et al., 2012), although not through regulation of AP-1 recruitment to the TGN. Whether either of the type II PI4Ks has a similar function in mammals is unknown, although our previous studies suggest that a role in WPB formation and function is likely. Our investigations reveal a crucial role for these kinases, in supporting the formation of WPBs, the ability of endothelial cells to produce pro-thrombotic VWF strings and to provide a fully functional haemostatic system in mice.

## RESULTS

### A TGN-located pool of PI4P can be detected in HUVECs

**Fig. 1.**
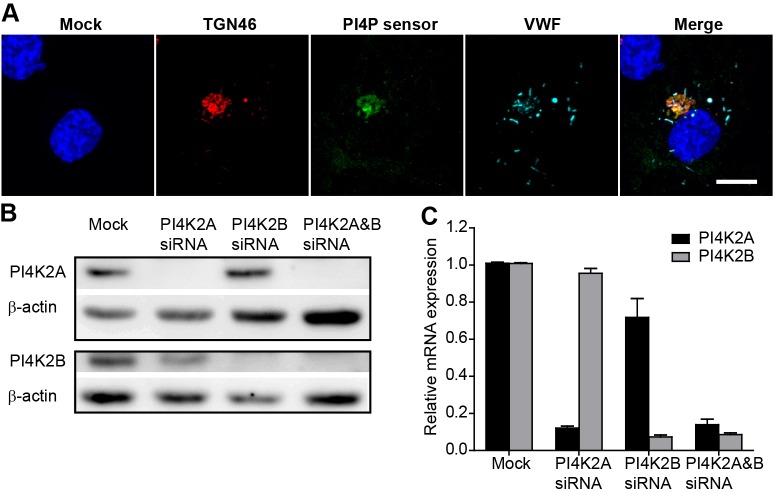
**Localisation of PI4P, and RNAi of PI4K2A and PI4K2B in endothelial cells.** (A) Representative confocal image of a HUVEC transfected with the PI4P probe GFP-SidC, fixed, permeabilised and labelled with DAPI nuclear stain (blue), anti-TGN46 (red), anti-GFP (green) and anti-VWF (cyan). Scale bar: 10 µm. HUVEC were transfected with vehicle (Mock), or siRNA against PI4K2A, PI4K2B or both PI4K2A and PI4K2B (PI4K2A&B). (B,C) The efficiency of knockdown was assayed by detecting protein levels (western blotting, B) or mRNA transcript levels (qRT-PCR; C). Means±s.e.m. of six experiments are shown in C.

To confirm the presence of a TGN-located pool of PI4P in human endothelial cells, we overexpressed a GFP-tagged version of the specific PI4P sensor protein SidC (GFP-SidC) (Luo et al., 2015) in human umbilical vein endothelial cells (HUVECs). The PI4P sensor primarily decorates a typical Golgi structure – overlapping with the TGN marker TGN46 – but not the adjacent WPBs (Fig. 1A), indicating high levels of the lipid at the TGN where WPBs are formed, but not on mature WPBs. The expression of the kinases PI4KIIα and PI4KIIβ in HUVECs was confirmed by western blotting (Fig. 1B) and quantitative real-time (qRT)-PCR (Fig. 1C), and each kinase can be specifically ablated by small interfering RNA (siRNA) (Fig. 1B,C).

### RNAi-meditated ablation of PI4KIIα and/or PI4KIIβ produces shorter WPBs with abnormally folded VWF

**Fig. 2.**
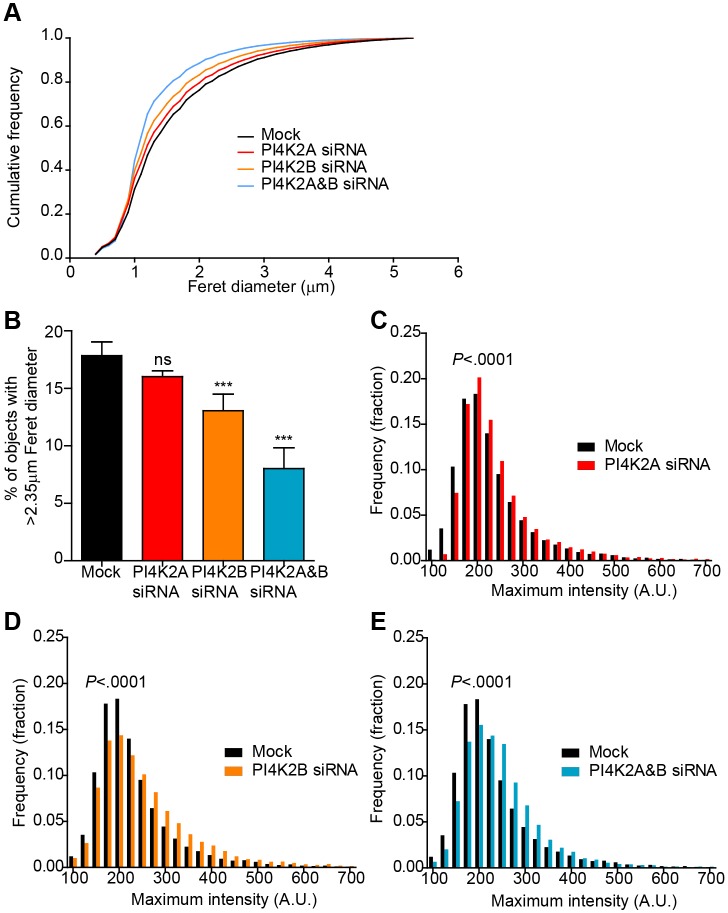
**Morphometric analysis of VWF-positive structures in PI4KII-depleted cells.** (A–C) The morphology of WPBs in HUVECs transfected with vehicle (Mock) or siRNA against PI4K2A, PI4K2B or both PI4K2A and PI4K2B (PI4K2A&B), was analysed by using an unbiased high-throughput method. The Feret diameter (A, B) and maximum fluorescence intensity (C-E) of VWF-positive objects in confocal images of HUVECs labelled with anti-VWF was measured. Graphs are representative of at least three determinations. In A, a cumulative frequency distribution representative of three determinations is shown. In each case, the results for the siRNA-treated groups were considered significant by Wilcoxon rank-sum test (*P*<0.0001). In B, the percentage of VWF-positive objects with a Feret diameter of >2.35 µm is shown (see Fig. S1). Means±s.e.m. of eight replicate wells, analysed by one-way ANOVA with Dunnett's multiple comparison test, compared to the mock sample. ns, non-significant; ***P<0.001. In C–E, frequency distributions of the maximum intensity values for each VWF-positive object are shown. Data were analysed by using the Wilcoxon rank-sum test. A. U, arbitrary units.

The elongated cigar-like shape of WPBs reflects the presence of VWF correctly folded into the tubules that are crucial to its function (Michaux et al., 2003). We have previously shown that interference with the protein machinery involved in early WPB formation at the TGN can alter their shape (Michaux et al., 2006a). We now find that confocal microscopy also suggests a change in the morphology of WPBs in PI4KIIα and/or PI4KIIβ depleted HUVECs. To confirm this, an unbiased automated high-throughput morphometric analysis was performed (Ferraro et al., 2014), where >10^5^ WPBs were analysed per condition (a detailed explanation of the rationale behind the presentation of this data can be found in Fig. S1). We find that the distribution of WPB length (Feret diameter) was significantly altered in all PI4KII kinase-depleted samples, showing an increase in the proportion of short relative to long VWF-positive objects (Fig. 2A). This was mirrored by a change in length when WPBs deficient in PI4KIIα were analysed by using electron microscopy (EM) (Fig. S2).

Whereas longer WPBs represent a minority of the total WPB population, they contain a disproportionally large amount of VWF (Ferraro et al., 2014) (Fig. S1) and also produce the largest and, thus most active VWF structures on release (Ferraro et al., 2014). We, therefore, examined the effects of loss of the PI4KII kinases specifically on the population of longer WPB, which corresponds to 50% of the total VWF area. We find that there are significantly fewer long WPBs when PI4KIIβ is ablated compared to the effect of suppressing PI4KIIα (Fig. 2B), and that loss of both kinases gives a greater effect than ablation of either alone, together giving a 55% decrease in the number of long WPBs. This suggests that PI4KIIα and PI4KIIβ are both important for early WPB formation and their combined effect is, at least, additive. We predicted that this dramatic decrease in the proportion of long WPBs can have deleterious effects on WPB function, on the basis of our previous findings that a 50% decrease in the number of long WPBs causes a catastrophic decrease in the number of VWF strings generated by endothelial cells (Ferraro et al., 2014). We, therefore, proceeded to investigate whether this is, indeed, the case.

**Fig. 3.**
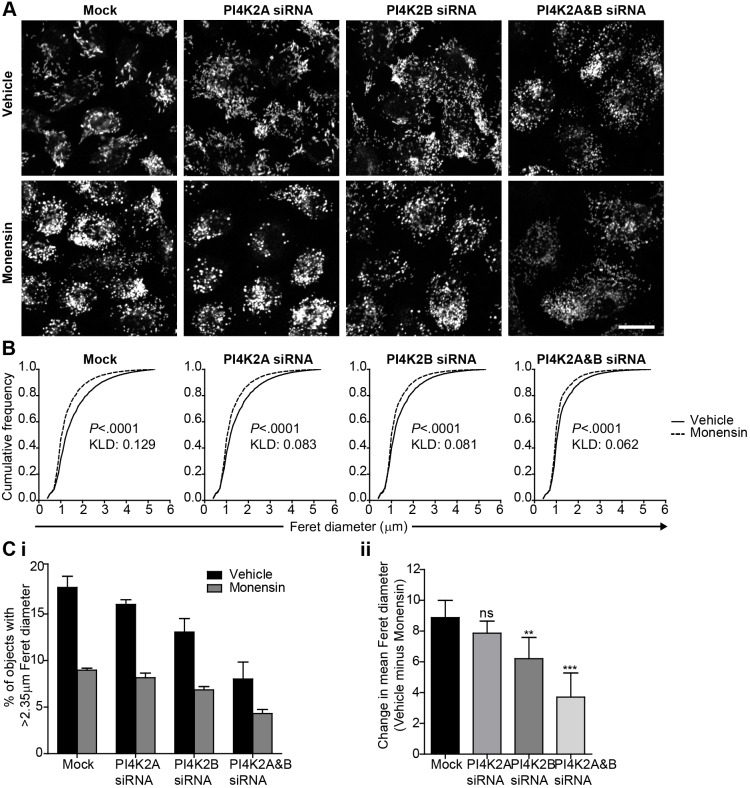
**Examination of VWF-positive objects after treatment with monensin.** HUVECs were transfected with vehicle (Mock) or siRNA against PI4K2A, PI4K2B or both transcripts, and treated with vehicle or monensin. (A) Opera acquired confocal sections showing VWF immunolabelling. Scale bar: 20 µm. (B) Cumulative frequency distributions of the Feret diameter of VWF-positive structures in cells treated with vehicle (line) or monensin (dotted line), together with results of Wilcoxon rank-sum test (*P* value) and Kulback–Leibler distance (KLD). Representative of three determinations. (C) Data from B displayed as a percentage of VWF-positive objects with a Feret diameter >2.35 µm (Ci) or the difference in mean Feret diameter between vehicle and monensin-treated samples (Cii). Means±95% confidence interval of eight replicate wells are shown. Data shown in Cii were analysed using one-way ANOVA with Dunnett's multiple comparison test compared to the mock sample. ns, non-significant; **P<0.01, ***P<0.001.

WPB shape depends to a large extent on the correct folding of VWF into proteinaceous tubules (Michaux et al., 2006a), so if depletion of the PI4KII isozymes causes a change in WPB shape, it is possible that the structure of VWF is altered within these PI4KII-deficient cells. Previously, we have observed that, following treatment with monensin, deliberate unfolding of VWF within WPBs causes a loss of propeptide binding and the consequent unfolding of intraorganellar VWF. This not only leads to their change from cigar-shaped to spherical organelles (Michaux et al., 2006a) but also increases the intensity of VWF staining in immunofluorescence analyses (Gregory Michaux and D.F.C., unpublished observation), presumably due to a higher accessibility of epitopes to the VWF antibody. If the defect in WPB shape, as seen in the PI4KII-depleted cells, partly reflects a failure to correctly pack VWF into WPBs, we hypothesise that WPBs in these cells display brighter VWF fluorescence intensity than WPBs in control cells. Moreover, monensin treatment should produce a significant decrease in the length of control WPBs as well as an increase in the staining intensity of VWF, but the magnitude of these effects should be less in PI4KII-deficient cells. Indeed, we found that WPBs in PI4KII-depleted cells are significantly brighter than WPBs in mock-treated cells (Fig. 2C–E), as shown by the shift of the frequency distribution towards a higher intensity. Images of these WPBs in HUVECs treated with vehicle or monesin are shown in Fig. 3, illustrating that – in monensin-treated cells – VWF-positive objects become smaller (Fig. 3A,B). Interestingly, the difference between the frequency distributions with and without monensin treatment is less in the PI4KII-depleted cells than in controls, as indicated by a decrease in the value for the Kullback–Leibler distance (KLD) (Fig. 3B). The dramatic effect of monensin on WPB length is exemplified by a significant decrease in the proportion of long (>2.35 μm) VWF-positive objects in all groups (Fig. 3Ci). Again, the magnitude of this shift is smaller in PI4KII-depleted cells (Fig. 3Cii). Taken together, these results point towards a defect in packing of VWF within WPBs in PI4KII-depleted cells.

### PI4KIIα-deficient mice display abnormal WPBs

**Fig. 4.**
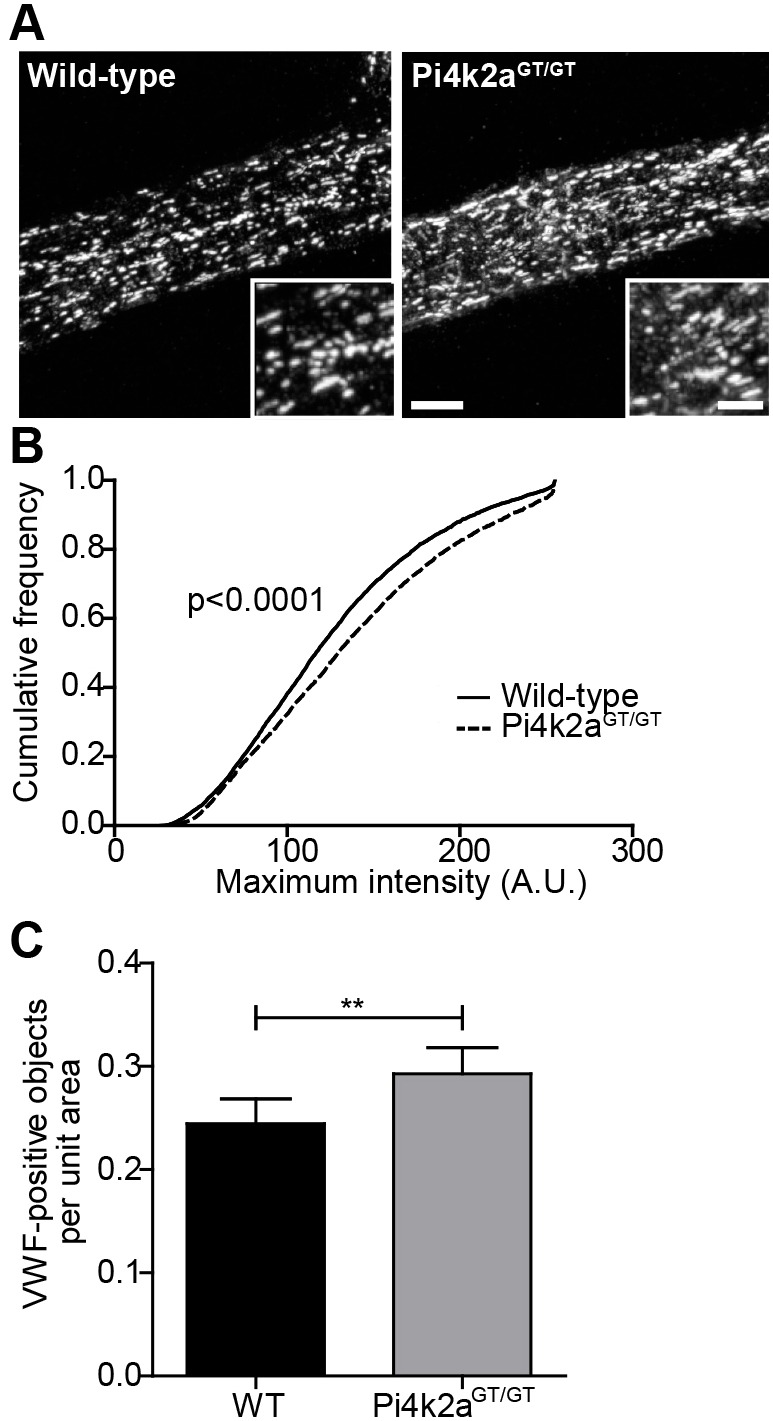
**Confocal analysis of WPBs in the retinal vasculature of PI4KIIα-deficient mice.** (A) Representative maximum intensity projections of retinal blood vessels from wild-type or Pi4k2a^GT/GT^ mice, showing VWF immunolabelling. Scale bars: 10 µm or 2 µm (insert). (B) Cumulative frequency distribution of the maximum intensity of VWF-positive objects in similar images from wild-type or Pi4k2a^GT/GT^ mice, analysed by Mann–Whitney test. (C) The number of VWF-positive objects per unit area analysed by unpaired *t*-test. **P<0.01. A.U., arbitrary units.

We have previously generated a gene-trapped mouse strain lacking PI4KIIα activity (Simons et al., 2009). These mice initially appear normal but develop late-onset spinocerebellar degeneration. To extend our *in vitro* observations we examined WPBs in the neuroretinal vasculature of these mice, as there is no known viable mouse model for PI4KIIβ deficiency. The retina is a readily accessible tissue that can be mounted as a flattened preparation, allowing some limited light-microscopic analyses of WPBs in a physiological context. Confocal imaging revealed the presence of a high number of WPBs within vessels of the superficial plexus and their alignment with the direction of flow in the superficial arterioles was clearly apparent (Fig. 4A). This is in contrast to reports on HUVECs grown under flow conditions, where no relation between the direction of flow and the orientation of the organelles was observed (Dragt et al., 2012). The parallel alignment of the WPBs should facilitate measurement of WPB Feret diameter from confocal images; however, we could not detect a change in WPB length within the retinal vasculature of PI4KIIα-deficient mice (data not shown). In contrast, when we examined the fluorescence intensity of the VWF-positive objects, we detected a significant increase in intensity in the endothelial cells of the PI4KIIα-deficient mice compared with littermate controls (Fig. 4B) that is mirrored by our *in vitro* data. Interestingly, there were more VWF-positive objects per unit area in the PI4KIIα-deficient mice compared to that of controls (Fig. 4C), which is different to our *in vitro* observations (data not shown).

### PI4KII-deficient HUVECs secrete normal levels of correctly multimerised VWF, but string formation is impaired and PI4KIIα-deficient mice exhibit a bleeding phenotype

**Fig. 5.**
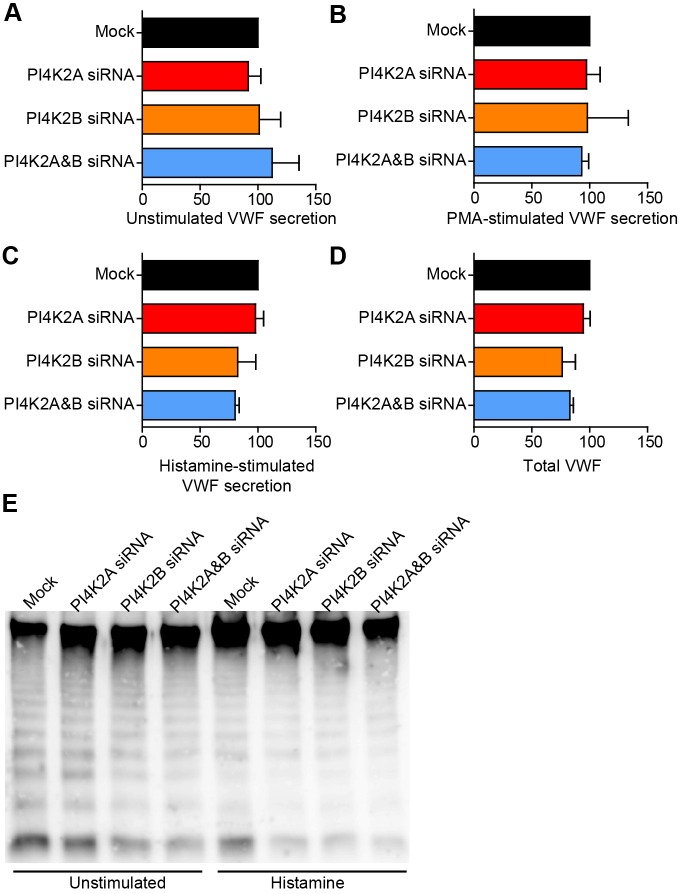
**Effect of PI4KII-kinase depletion on secreted VWF protein.** (A–C) HUVECs treated with vehicle (Mock) or siRNA against PI4K2A, PI4K2B or both transcripts were either not stimulated (A), stimulated with PMA (B) or with histamine (C). Secreted VWF (A–C) and total VWF levels (D) were measured by ELISA and were typically 3% (±0.6%) (unstimulated), 10% (±1.2%) (histamine) and 24% (±2.9%) (PMA) of total VWF content. Here secreted VWF is presented as a percentage of mock values. Means±s.e.m. of three to six experiments are shown and were not significantly different (analysed using one-way ANOVA). (E) Secreted VWF from unstimulated (60 min) or histamine-stimulated (30 min) HUVECs was analysed for VWF multimer distribution on a non-reducing multimer gel. No differences were observed between samples.

VWF function in haemostasis depends not only on the amount released but also on its structure (Nightingale et al., 2009). Despite the clear effects of PI4KII-deficiency on WPB morphology and folding of their VWF content, no quantitative defect in VWF secretion was found, which is consistent with previous results (Michaux et al., 2006a). RNA interference (RNAi)-mediated ablation of either or both kinases did not alter the amount of VWF released from unstimulated HUVECs (Fig. 5A). Moreover, the levels of VWF secreted in response to the protein kinase C activator phorbol 12-myristate 13-acetate (PMA) (Fig. 5B) or the physiological agonist histamine (Fig. 5C) were comparable to control HUVECs, and total VWF content was largely unchanged (Fig. 5D).

VWF multimers of higher molecular weight adhere better to collagen and platelets (Federici et al., 1989) and are, thus, more effective at supporting thrombus formation in conditions of high shear stress. We next examined the multimeric state of VWF secreted from PI4KII-deficient HUVECs and, again, detected no differences between control and kinase-deficient samples (Fig. 5E), suggesting that the ability of VWF to multimerise remains unaffected in these PI4KII-deficient cells. However, because an important role of WPBs during haemostasis is the production of long VWF-strings on the surface of endothelial cells for platelet capture (Michaux et al., 2006a; Valentijn and Eikenboom, 2013) and because short WPB can give altered string production without affecting VWF multimerisation (Ferraro et al., 2014), we postulated that the effect of the alterations in VWF structure that is reflected in the changes in WPB shape becomes apparent when partially misfolded VWF filaments unfurl and combine during string formation. We, therefore, examined the ability of PI4KII-deficient HUVECs to produce and release VWF strings.

**Fig. 6.**
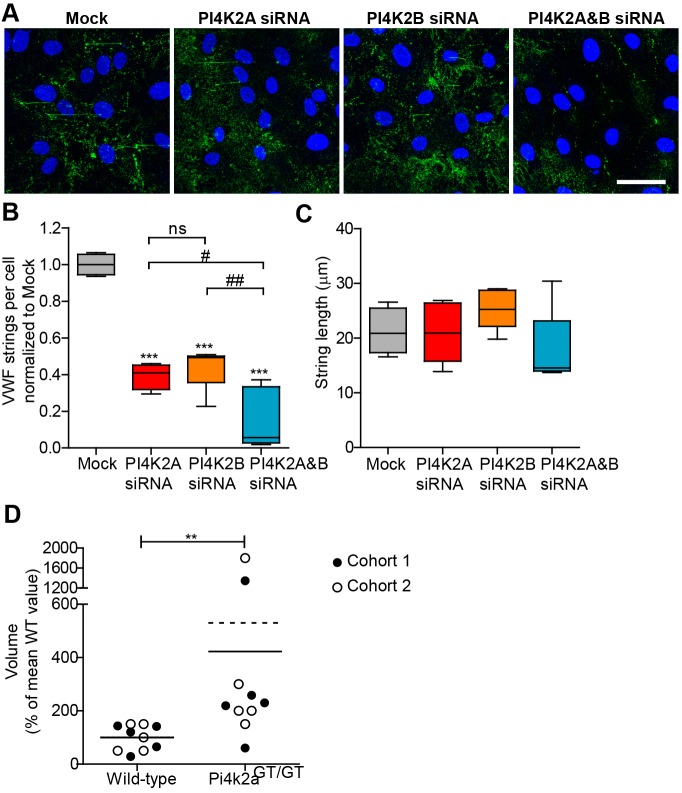
**Effect of PI4KII-kinase depletion on VWF string formation *in vitro* and bleeding time *in vivo*.** (A) HUVECs treated with vehicle (Mock) or siRNA against PI4K2A, PI4K2B or both transcripts (PI4K2A&B) were stimulated with histamine under flow to release VWF strings, then fixed under flow and their surface was labelled with anti-VWF for imaging. Scale bar: 50μm. (B,C) String number per cell (B) and string length (C), were then measured, with whisker plot showing mean, maximum and minimum of more than four experiments. Data were analysed by one-way ANOVA (*P*<0.0001) and Tukey's multiple comparison post-test employed to compare all samples to mock (***P<0.001), or between siRNA-treated groups as indicated (not significant (ns), ^#^*P*<0.05, ^##^*P*<0.01). (D) Blood loss volume following tail cut from Pi4k2a^GT/GT^ mice from two cohorts of five mice per condition was measured. Each dot represents a single mouse. Lines represent the mean from each independent cohort. Data were analysed by Wilcoxon rank-sum test. **P<0.05. Removal of outliers (bleeding time >1200 s) from the analysis resulted in **P<0.01.

Mock and siRNA-treated HUVEC were placed under flow, treated with histamine to stimulate WPB exocytosis and then fixed under flow to maintain the integrity of the VWF strings produced. Cells were then surface stained with anti-VWF and processed for image acquisition (Fig. 6A). A dramatic reduction in the mean number of VWF strings, of 60% (±7%) for PI4KIIα and 56% (±12%) for PI4KIIβ, when compared to controls was observed (Fig. 6B). Ablation of both enzymes together is significantly more effective than either kinase alone, reducing VWF string number by 84% (±16%). Of note, the mean length of the few VWF strings measured in all of the siRNA-treated cells was similar (Fig. 6C) which, combined with the reduction in string number, is a previously unknown phenotype. Together, these results suggest that these closely related kinases have overlapping but non-redundant functions in WPB biogenesis and VWF string formation.

Finally, we investigated the possibility that the degree of string loss observed *in vitro* is accompanied by a phenotype of excessive bleeding *in vivo*. To investigate the effect of PI4KII-deficiency on haemostasis, blood loss volume in Pi4k2a^GT/GT^ and control mice was measured after amputating the tail tip of the mice (Fig. 6D). Two cohorts of mice, totalling ten mice per group, were independently analysed. In each case, the volume of blood lost after 7 min was significantly greater in PI4KIIα-deficient mice, suggesting altered haemostasis in the absence of PI4KIIα.

## DISCUSSION

WPB biogenesis and the structural processing of VWF are interdependent processes that underpin the availability and function of WPB cargo, which can be physiologically modulated or affected by disease. The data presented here establish a regulatory role for two lipid kinases, PI4KIIα and PI4KIIβ, in these processes. We propose that production of PI4P at the TGN through these isozymes can orchestrate the recruitment of proteins and lipids involved in WPB formation. Ablating the expression of PI4KIIα and PI4KIIβ in primary endothelial cells results in a so-far-unknown and complex phenotype; a change in WPB morphology and internal VWF structure and, despite normal levels of VWF secretion, a massively reduced number of VWF strings released under flow upon endothelial activation. In addition, mice deficient in PI4KIIα, have abnormal WPBs and impaired haemostasis, which mirrors and extends our *in vitro* data.

The deliberate reduction of VWF expression can produce fewer and shorter WPBs (Ferraro et al., 2014). However, in this case siRNA-mediated ablation of PI4KIIα and PI4KIIβ does not affect the total amount of VWF within cells or secreted by cells, and the WPB number per cell is not altered *in vitro*. Yet, when we examined the shape of these WPB by using an unbiased high-throughput automated method, we discovered that, generally, WPBs were significantly shorter. This was most apparent when the functionally more significant ‘long’ WPBs were examined; we observed a 60% loss of WPBs longer than 2.35 μm when both isozymes were ablated. Although we could not reliably measure WPB length *in vivo*, we did observe a greater density of WPBs in the neuroretinal endothelium of PI4KIIα-deficient mice compared with those of littermate controls, implying an alternative regulation of the relationship between number and size of WPBs. Whether this reflects a difference between the *in vitro* and *in situ* situation of species or of tissues is unclear. However, given the complexities that our detailed analyses are revealing, such differences are not surprising.

We have previously shown that treatment of HUVECs with monensin or chloroquine, to neutralise the pH of acidic organelles, causes a loss of VWF tubules and shortening of WPBs (Michaux et al., 2006a). We hypothesised that, if VWF tubulation is affected in PI4KII-depleted cells, monensin should have a reduced effect on the WPB of PI4KII-depleted cells compared to controls. Our unbiased high-throughput approach confirmed this. Moreover, in cells where PI4KIIα and PI4KIIβ are both ablated and WPBs are severely abnormal, monensin still shortens the WPBs, suggesting some retention of tubular coiling. It is notable that these WPBs – like monensin- or chloroquine-treated WPBs (Michaux et al., 2006a) – respond normally to secretagogue stimulation. Together, these data strongly suggest that the loss of PI4KIIα or PI4KIIβ affects the intra-organellar milieu of the WPB, impacting on VWF string structure.

Further analysis showed that WPBs in PI4KII-deficient HUVECs also show an increase in VWF antibody binding. This change was significant in both the PI4KIIα- and PI4KIIβ-ablated cells but each phenotype was, again, exacerbated when both kinases were suppressed. We propose that the relative intensity of VWF staining can be used to measure tubule integrity or packing within WPBs, and that the VWF in WPBs of PI4KII-deficient HUVECs is not as tightly-packed as in controls (Geysen et al., 1987). Importantly, the data obtained by using intensity measurements from PI4KIIα-deficient mice are in agreement with the *in vitro* data and further demonstrate that WPBs are abnormal in these mice. Moreover, the phenotype of excessive bleeding observed in these mice is consistent with this defect in WPB formation.

The general technique to investigate WPB defects is the enzyme-linked immunosorbent assay (ELISA) for the detection of VWF release, which can be used as a reporter of both defective WPB formation and of exocytic failure. In this study, we did not see any secretory phenotype in the siRNA-treated cells when carrying out VWF ELISA. However, the production of extruded VWF strings is affected by several other factors, including the length of WPBs (Ferraro et al., 2014) and VWF tubulation (Michaux et al., 2006a). Both of these factors are affected here, and analysis under flow revealed a dramatic fall in the number of VWF strings in the PI4KII-depleted cells, with the double knockdown cells, again, showing the biggest change. Shorter WPBs generated by fragmenting the Golgi complex can cause shorter strings to be formed (Ferraro et al., 2014); however, here, shorter WPBs are not associated with shorter VWF strings but, rather, with a decrease in string number. The magnitude of this decrease in the double knockdown suggests that in a 100% efficient treatment with siRNA there may be no strings formed at all. Thus, a different and, potentially, unknown mechanism is likely to be in play and warrants further investigation.

PI4KIIα is reported to be the prominent regulator of PI4P formation at the TGN and is constitutively resident at the TGN and endosomal boundary (Simons et al., 2009; Wang et al., 2003). PI4KIIα can associate with adaptor proteins AP-1 (Wang et al., 2003) and AP-3 (Craige et al., 2008), and with Hermansky–Pudlak syndrome protein complexes (Salazar et al., 2009), and is important for regulation of receptor tyrosine kinase (Minogue et al., 2006) and Wnt signalling (Mossinger et al., 2012; Pan et al., 2008; Qin et al., 2009). Less is known about its close relative PI4KIIβ, a partially cytosolic enzyme that is believed to associate with membranes in response to stimuli such as growth factor (Wei et al., 2002), and which may associate with clathrin coated vesicles (Li et al., 2012). How the loss of these enzymes causes the alterations in WPB length and VWF folding or packing is still unclear, but the fact that kinase ablation does not affect the amount of VWF produced or secreted suggests that their primary affects are at the TGN, modulating initial WPB formation. Since HPS6, a subunit of the BLOC-2 complex, is needed for VWF exocytosis from HUVECs (Sharda et al., 2015), and the related BLOC-1 complex binds to PI4KIIa in association with AP-3 (Salazar et al., 2009) in a complex that also associates with BLOC2, we cannot rule out other explanations, such as a more indirect effect involving this endosome-associated complex.

One possibility is that loss of type II PI4Ks reduces recruitment of AP-1 to the TGN. To our surprise, we did not see an AP-1-deficiency phenotype, i.e. formation of small VWF-positive punctae and loss of regulated secretion (Lui-Roberts et al., 2005). However, PI4P has been reported to induce curvature in model membranes at physiologically relevant concentrations (Furse et al., 2012) and can regulate the recruitment of other PI4P-binding proteins, including the Golgi-localising, gamma-adaptin ear-domain homology, ARF-binding (GGA) adaptor proteins (Wang et al., 2007) and lipid-transfer proteins that include oxysterol-binding protein (OSBP), ceramide transfer (CERT) and four-phosphate-adaptor (FAPP) proteins (Banerji et al., 2010; Hanada et al., 2003; Liu et al., 2014), which facilitate changes in membrane composition and shape through curvature-inducing domains (e.g. epsin N-terminal homology domains) and by directing coat assembly, or by generating membrane asymmetry (Natarajan et al., 2009). Identifying which of these downstream players are important to WPB formation is part of our future studies.

We present new tools to detect and analyse defects in WPB biogenesis and VWF structure, for example, the use of monensin and antibody accessibility to unravel VWF folding or packing. Unbiased high-throughput automated analysis enables quantification of large numbers of WPBs and reveals morphological differences that, otherwise, might well be missed.

Together, this first demonstration of PI kinases being important for the formation and function of a regulated secretory organelle in mammals highlights the importance of lipid-modifying enzymes as cellular controls on WPB formation. We suspect that this is only the first report of analyses that might ultimately reveal the role of different kinases in controlling a number of different steps that underpin VWF function.

## MATERIALS AND METHODS

### Antibodies

Rabbit polyclonal anti-VWF used for ELISA (dilution 1:600), multimer gel (dilution 1:4000) and extracellular string analysis (dilution 1:10,000) was from DakoCytomation. VWF-HRP-conjugated antibody used for ELISA (1:1000) was from DakoCytomation. Sheep polyclonal anti-VWF (dilution 1:1000) and anti-TGN46 (dilution 1:500) used for Immunofluorescence were from Serotec. For high-throughput morphological analysis, a rabbit antibody targeting the pro-region of VWF was used (dilution 1:1000) (Hewlett et al., 2011). Alexa-Fluor(-488, -594 or -647)-conjugated secondary antibodies (all used at 1:500 dilution) were from Life Technologies. Anti-PI4KIIα and anti-PI4KIIβ antibodies for western blotting (dilutions 1:2000 and 1:250 respectively) have been previously described (Mossinger et al., 2012). Anti-β-actin was from Santa Cruz (dilution 1:500). Rabbit and mouse HRP-conjugated secondary antibodies were from Jackson ImmunoResearch Laboratories (dilutions 1:500 and 1:3000, respectively).

### Tissue culture and transfection

HUVECs were cultured and transfected as described previously (Michaux et al., 2006b). To minimise effector-binding inhibition artifacts the GFP-SidC construct (Luo et al., 2015) was expressed for just 20 h and only medium- to low-expressing cells were analysed. For monensin treatment, HUVECs were incubated with 10 μM monensin for 1 h at 37°C just before processing for immunofluorescence.

### RNAi and nucleofection

PI4KIIα and PI4KIIβ depletion was achieved by using siRNAs with the sequence 5′-GGAUCAUUGCUGUCUUCAA(TT)-3′ and 5′-GGUUCAAGUGGAAGUUACU(TT)-3′ (Eurofins/MWG), respectively. Mock transfections were performed in parallel without siRNA. HUVECs underwent two rounds of transfection with 300 pmol of siRNA per nucleofection (Amaxa Biosystems) as described previously (Lui-Roberts et al., 2008). Cells were assayed 2 days following the second round of nucleofection at 100% confluency.

### Real-time qPCR

At the end of each experiment HUVECs were collected for RNA extraction, cDNA preparation and quantitative real-time (qRT)-PCR as previously described (Nightingale et al., 2009). Gene expression was quantified by the 2^−ΔΔC(T)^ method (Livak and Schmittgen, 2001) normalised with a β-actin internal control. The primers used for β-actin amplification were 5′-TGGTGGTGAAGCTGTAGCC-3′ and 5′-GCGAGAAGATGACCCAGAT-3′. For PI4K2A, the primers 5′-ATTGCCTCGATCAGTGTTGC-3′ and 5′-ACCAAAGGTTGGTTCATTCC-3′ were used. For PI4K2B, a Qiagen QuantiTect primer assay (catalogue number QT00059549) was used.

### VWF secretion assays

The VWF secretion assay has been described previously (Lui-Roberts et al., 2005). In short, cells were rinsed and incubated in serum-free medium for 30 min for collection of unstimulated secretion. The medium was then collected and replaced with fresh medium containing secretagogues, either 100 ng/ml phorbol 12-myristate 13-acetate (PMA) or 10 μM histamine. The medium containing the released VWF was collected after 30 min and the remaining cells were lysed to determine total VWF levels. The relative amounts of VWF were quantified by enzyme-linked immunosorbent assay (ELISA) (Blagoveshchenskaya et al., 2002), and the data were normalised by using the total VWF signal. The secretagogue-responsive pool was estimated by subtracting the amount of VWF released by unstimulated secretion from that released upon secretagogue addition. To combine data from various independent experiments, data were normalised to the mock samples in each experiment.

### VWF multimer gel

Secreted VWF from unstimulated and 10 μM histamine-stimulated HUVECs was analysed as described previously (Lui-Roberts et al., 2008). Briefly, secretion assay samples were loaded onto 1.4% agarose SDS-gels to separate the multimers, then transferred to a nitrocellulose membrane and labelled with anti-VWF antibody followed by a HRP-conjugated secondary antibody.

### Cell immunofluorescence and image acquisition

Cells were fixed, prepared as described previously (Lui-Roberts et al., 2005) and examined at ambient temperature through a 63× oil immersion lens (NA 1.3) on a TCS SP5 confocal microscope system (Leica, Wetzlar, Germany). Adobe Photoshop 6.0.1, Illustrator CS5 (Adobe Systems, Mountain View, CA) or ImageJ (Schneider et al., 2012) were used to generate figures from digital images.

Image processing and parameters used for high-throughput morphological analysis are described in detail elsewhere (Ferraro et al., 2014). In brief, images were acquired using an Opera high-content screening system (Perkin Elmer) by using a 40× air objective (NA 0.6). At least eight wells per condition were analysed per experiment, with nine images per well. Maximum Feret diameter (also known as maximum caliper) and maximum intensity of each VWF-positive object was measured. At least 100,000 VWF-positive structures were analysed per condition. To select the population of longer WPBs in an unbiased fashion for each control sample in each individual experiment, VWF-positive objects were sorted by Feret diameter and the cumulative area was calculated. The top fraction by size of objects that contained half of the cumulative area were chosen, and the corresponding Feret diameter of this cut off, typically 2.3–2.4 μm, was used for all samples within the same experiment. The fraction of VWF-positive objects with a Feret diameter longer than this cut-off was calculated and plotted as a proportion of the total population.

### *In situ* and *in vivo* studies

Pi4k2a^GT/GT^ mice, which lack PI4KIIα kinase activity, were generated as described previously (Simons et al., 2009). Retinal whole-mounts from two cohorts of ten animals (five WT littermate controls and five Pi4k2a^GT/GT^ mice) of 6 months of age were fixed and stained as previously described (Fruttiger, 2002) with antibodies against VWF and CD31. *Z*-stacks of superficial arteries with similar diameters were acquired at ambient temperature through a 63× oil immersion lens (NA 1.3) on a TCS SPE confocal microscope system (Leica, Wetzlar, Germany). Analysis of the maximum intensity of VWF-positive objects was performed on six maximum-intensity projections (MIPs) from each mouse using ImageJ. In order to extract the outline of a hemi-vessel, the MIP image was generated on the endothelial marker (CD31) channel image stack. The VWF channel was then segmented by using a manually chosen threshold value. Watershed segmentation was applied in parallel on a copy of the original MIP image of the VWF channel with a value of 50 as noise tolerance parameter. The resulted ‘mesh’ image of the watershed-segmented WPB particles was used to separate the merged WPBs on the other, manually thresholded, binary MIP image of the VWF channel. WPB Feret diameter, area, mean grey value and total intensity were selected as features to measure. WPB objects between area 0.09–8.00 μm^2^ were measured, the resulting table was saved in comma-separated file format for further analysis. For quality control, the region of interest set of all WPB segmentation outlines was saved and an outline image was generated for each VWF MIP image in order to validate the precision of the image analysis workflow.

Tail-bleeding assays were performed by measuring the volume of blood in a defined period of time (7 min) in a manner similar to that described previously (Liu et al., 2012), except that the blood was collected at room temperature directly into a pre-weighed tube and the blood volume calculated by re-weighing the tube. Animal studies were ethically reviewed and approved by the UCL Royal Free Campus Ethics and Welfare Committee and the UK Home Office, and complied fully with European Directive 86/609/EEC.

### VWF string assay

Nucleofected HUVECs were seeded onto gelatin-coated µ-slides VI0.4 (ibidi, Munich, Germany). Slides were mounted on the microscope stage of an Axiovert 100 (Carl Zeiss, Welwyn Garden City, UK), maintained at 37°C, and connected to a syringe pump system (Harvard Apparatus, Holliston, MA) to draw fluid through the chamber with a wall shear stress of 25 Pa (2.5 dyne/cm^2^). Cells were rinsed with perfusion medium (HBSS containing Ca^2+^ and Mg^2+^, and supplemented with 0.2% BSA) under flow, to flush through cellular debris and ensure the HUVEC monolayer was intact. Cells were then perfused with perfusion medium±10 μM histamine for 5 min followed by perfusion with 4% PFA. Flow speed was gradually decreased until it stopped completely and cells were left in the presence of PFA for a total of 20 min, followed by washing and processing for immunofluorescence without permeabilisation, in order to only stain VWF on the surface of cells with rabbit anti-VWF antibody and nuclei with DAPI. Thirty fields of view were taken for each sample at ambient temperature by using a 20× oil objective on a Leica SP5 confocal scanning microscope covering an area of 4.5 mm^2^ (∼500–900 nuclei). The length of each VWF string was measured manually by using ImageJ.

### Statistics

For high-throughput morphometry, all statistical analysis was done using R (http://www.r-project.org/). Nonparametric, two-tailed, two-independent-sample Wilcoxon rank-sum test was used to determine statistical significance of changes in Feret diameter and maximum intensity. Kullback–Leibler distance was used to estimate the extent of change between cumulative frequency curves. All other statistical analysis was done by using GraphPad Prism version 6.0 software, using the tests indicated in the associated figure legends. Error bars indicate the mean±95% confidence interval except where indicated otherwise.
